# An Improved SBAS-InSAR Processing Method Considering Phase Consistency: Application to Landslide Monitoring in Hualong County, Qinghai Province, China

**DOI:** 10.3390/s26020420

**Published:** 2026-01-08

**Authors:** Wulinhong Luo, Bo Liu, Guangcai Feng, Zhiqiang Xiong, Wei Yin, Haiyan Wang, You Yu, Peiyu Chen, Jixiong Yang

**Affiliations:** 1 School of Geosciences and Info-Physics, Central South University, Changsha 410083, China; luowulinhong@csu.edu.cn (W.L.); zqxiong_flhs@csu.edu.cn (Z.X.);; 2 The Geohazards Survey and Monitor Institute of Hunan Province, Changsha 410029, China; 3 The Hunan Geological Disaster Monitoring, Early Warning and Emergency Rescue Engineering Technology Research Center, Changsha 410029, China; 4 The Chongqing 208 Geological Environment Research Institute Company, Ltd., Chongqing 400799, China; wanghaiyancs@163.com

**Keywords:** Small Baseline Subset Interferometric Synthetic Aperture Radar (SBAS-InSAR), Nonzero Closure Phase (NCP), landslide detect and monitoring, phase consistency

## Abstract

Phase consistency is a critical prerequisite for achieving high-precision time-series InSAR deformation retrieval. However, conventional SBAS-InSAR methods provide only limited consideration of phase consistency during the inversion process. Within the SBAS-InSAR workflow, two principal categories of error sources are primarily responsible for phase inconsistency, manifested as non-zero closure phase (NCP): (1) fading biases introduced during multilooking and filtering prior to phase unwrapping; and (2) unwrapping errors caused by large deformation gradients, low coherence, or inappropriate selection of unwrapping algorithms. To address these issues, this study introduces an improved SBAS-InSAR processing workflow, termed NCP-SBAS, designed to improve the accuracy of deformation field estimation and thereby enhance its applicability to geological hazard monitoring. The key idea of the method is to enforce phase consistency as a constraint, jointly accounting for the spatiotemporal characteristics of fading biases and the valid deformation signals, thereby enabling effective correction of NCP. To evaluate the effectiveness of NCP-SBAS, this study conducted a detailed analysis of deformation differences in Hualong County, Qinghai Province, before and after NCP correction, highlighting the significant advantages of the proposed approach. The results indicate that the influence of fading biases on deformation estimates depends on both the magnitude and direction of deformation, while unwrapping errors primarily lead to an underestimation of deformation. In addition, the study provides an in-depth discussion of how fading biases and unwrapping errors affect landslide monitoring and identification.

## 1. Introduction

Multi-temporal Interferometric Synthetic Aperture Radar (MT-InSAR) has become one of the most powerful tools for retrieving high-precision deformation fields and has been widely employed in the monitoring of geological hazards, such as landslides, land subsidence, and mining deformation [1,2,3,4,5]. By exploiting stacks of interferograms, MT-InSAR enables the extraction of long-term, spatially dense deformation time series, providing valuable insights into hazard evolution. Commonly used MT-InSAR approaches include Persistent Scatterer InSAR (PS-InSAR), Small Baseline Subset InSAR (SBAS-InSAR), SqueeSAR, and StaMPS [6,7,8,9,10]. Among these, SBAS-InSAR, which relies on distributed scatterers (DS) and time series inversion of unwrapped interferograms, has become one of the most widely applied methods for geological hazard monitoring [11,12,13,14].

Effective error correction is a prerequisite for obtaining reliable deformation fields. In conventional SBAS-InSAR workflows, well-established methods exist for mitigating error components with distinct spatial low-pass characteristics, such as topographic residuals, orbital inaccuracies, and atmospheric delays. However, temporal error sources that compromise phase consistency have received comparatively little attention. Phase consistency is fundamental for robust phase estimation and accurate time series inversion in MT-InSAR [10,15,16]. According to the phase closure principle, the theoretical sum of phase differences around a closed loop of three or more interferograms should equal zero. Yet, in practice, two major error sources disrupt this principle: (1) fading bias introduced by multilooking and filtering operations; and (2) phase unwrapping errors caused by large deformation gradients, decorrelation, and algorithmic limitations. These errors give rise to nonzero closure phase (NCP) both before and after unwrapping, which accumulates along the temporal baseline and ultimately degrades deformation accuracy.

Recent studies have proposed a variety of strategies to mitigate fading bias. SBAS-InSAR commonly applies multilooking and filtering to enhance interferometric phase quality, but this inevitably sacrifices spatial resolution and mixes signals from scatterers with inconsistent temporal behavior, introducing fading bias [17]. To reduce such effects, closure triplets have been used to build observation matrices, with singular value decomposition (SVD) employed to estimate interferogram-specific biases [18]. However, when the number of closure loops is smaller than that of interferograms, the inversion becomes underdetermined, leading to large uncertainties. To overcome this, some approaches introduce linear dependence assumptions among closure loops with different temporal baselines [19], while others apply averaging strategies based on the law of large numbers [20]. More advanced techniques model amplitude evolution across long-span closure stacks [21] or exploit time-invariant and time-variant fading models with temporal windowing [22]. Despite these developments, existing methods either rely on oversimplified assumptions or fail to capture localized and spatially heterogeneous bias characteristics.

Similarly, unwrapping errors represent another critical challenge. Phase unwrapping aims to reconstruct continuous phase values from wrapped observations in the interval [−π, π], enabling deformation retrieval [23]. In theory, correctly unwrapped interferograms forming closed loops should yield zero closure phase. Thus, NCP serves as a sensitive indicator of unwrapping errors [24,25], particularly in two-dimensional algorithms, where NCP reflects temporal–spatial consistency. To correct such errors, two main categories of approaches have been developed. The first focuses on global correction via observation matrices and least-squares inversion, with regularization strategies such as L1-norm or Tikhonov frameworks applied to improve stability [26,27]. The second emphasizes localized corrections, integrating phase closure with morphological and re-estimation techniques [28]. However, these methods become ineffective when multiple interferograms within a closure loop simultaneously contain errors, leading to compounded ambiguities. To address this issue, iterative correction frameworks combining phase gradients, closure phases, and spatial coherence have been introduced [29]. While effective for large-scale regions, their applicability to small-scale hazards, such as localized landslides, remains limited due to mismatched detection window sizes.

To address the two aforementioned sources of error, this study proposes an improved SBAS-InSAR processing approach that explicitly incorporates the principle of phase consistency. By leveraging NCP derived from closure loops both before and after unwrapping, we aim to generate more accurate deformation fields and quantitatively assess the impacts of phase errors on geological hazard monitoring.

The remainder of this paper is organized as follows. Section 2 introduces the methodology for fading bias and unwrapping error correction. Section 3 presents the datasets and experimental results. Section 4 provides further discussion. Finally, Section 5 concludes the paper.

## 2. Methods and Data Processing

The proposed SBAS-InSAR workflow in GAMMA (version 2.9) includes data pre-processing, multilooking, spatiotemporal network construction, differential interferometry, phase unwrapping via the Minimum Cost Flow algorithm, orbital and atmospheric corrections, and time-series inversion using SVD. Two additional modules: (1) fading bias correction and (2) pixel-wise unwrapping error correction in deformation zones, are integrated into the standard pipeline, as detailed below.

### 2.1. Triplet Phase Closure

Assuming a dataset of N SAR acquisitions, a spatiotemporal baseline network can be constructed to generate M interferograms, with the interferometric phases before and after unwrapping denoted as $\left[\Delta \phi_{1,2},{\Delta \phi }_{1,3},\cdots ,\Delta \phi_{i,j}\right]$ and $\left[{\Delta \varphi }_{1,2},\Delta \varphi_{1,3},\cdots ,{\Delta \varphi }_{i,j}\right]$, respectively. Let i,j denote the acquisition times of the master and slave images. Select three interferograms that form a closed loop in time: $t_{h,k},\;t_{k,q},\;t_{h,q}$. Ideally, the triplet phase closure (TPC) should be zero both before and after phase unwrapping [10,15,16], as shown in Equations (1) and (2).

(1) $$D_{tpc}^{w}=F({\left\{\Delta \phi_{h,q}-\Delta \phi_{h,k}-\Delta \phi_{k,q}\right\}}_{i=1}^{n})$$

(2) $$D_{tpc}^{unw}=F({\left\{\Delta \varphi_{h,q}-\Delta \varphi_{h,k}-\Delta \varphi_{k,q}\right\}}_{i=1}^{n})$$

Fading bias and unwrapping errors both disrupt phase consistency, producing nonzero closure phases. In the wrapped phase, fading bias $D_{tpc}^{w}$ dominates the deviation, while in the unwrapped phase, both fading bias and unwrapping error, highlighting the importance of correcting fading bias. Hereafter, closure phases due to multilooking and filtering are denoted NCPw, and those from unwrapping errors as NCPunw.

### 2.2. Fading Bias Correction

Fading bias refers to phase deviations introduced by temporally inconsistent scattering behavior of multiple scatterers within a pixel, typically after multilooking and filtering. Its spatiotemporal characteristics are closely related to changes in surface scattering mechanisms. Intensity, a direct indicator of backscattering strength, effectively reflects regional scattering variations. For example, urban areas with stable scatters usually exhibit lower fading bias than agricultural areas, where soil moisture fluctuations result in higher temporal decorrelation [30,31]. Several studies have validated the effectiveness of amplitude-based fading bias correction methods [16,21]. Coherence, a key parameter for evaluating interferometric phase quality, is also closely associated with fading bias, determining its spatial distribution [30]. These observations suggest that both amplitude variation and coherence are important indicators of fading bias behavior. Moreover, fading bias demonstrates temporal decay characteristics, with stronger effects typically observed at shorter temporal baselines [19]. In this study, we account for the spatiotemporal behavior of fading bias in the correction process. The detailed correction steps are presented below.

#### 2.2.1. Calculation of ∆NCPw, ∆ins, and ∆coh

Fading bias is sensitive to both spatial averaging and temporal decorrelation effects introduced by multilooking and filtering. All possible triangle loops are iterated for each pixel, and the ∆NCPw, ∆ins, and ∆coh are computed accordingly. For both multilook ratios of 10:2 and 5:1, the wrapped phase, amplitude values, and coherence values are extracted. The difference between multilook ratios 5:1 and 10:2 captures the sensitivity of NCPw to fading bias while preserving fine-scale deformation. Using Equation (1), the NCPw is calculated. To avoid the suppression of subtle deformation signals that may arise from excessively large multilooking windows, this study employs multilook ratios of 5:1 and 10:2. These configurations achieve a balanced trade-off between noise reduction and the preservation of fine-scale deformation information, ensuring that the subsequent closure-phase analysis retains both spatial detail and phase stability.

To address the attenuation of fading bias, temporal baselines of the interferograms were incorporated into the calculation of intensity and coherence variations. The changes in fading bias, intensity, and coherence were then computed using Equations (3)–(5). Consider a set of three interferograms composed of master-slave pairs ab, bc, and ac, forming a triangle loop. The key parameters are defined as follows: the closure phase $\varphi_{NCPw}^{m}$ and the change in intensity $\rho_{ij}^{m}$ and coherence ${cc}_{ij}^{m}$ of each interferogram (i,j), where m denotes the multilook ratio. The temporal baseline for interferogram (i,j) is denoted by ${∆t}_{ij}$. The relative contributions of intensity (∆ins) and coherence variations (∆coh) to fading bias were quantified. Due to heterogeneous deformation patterns and differential ground target composition across multilook configurations, the sensitivity of NCPw to these variations exhibited notable regional differences.

(3) $${∆NCP}_{w}=\varphi_{NCPw}^{51}-\varphi_{NCPw}^{102}$$

(4) $$∆ins=\left[\left(\rho_{ab}^{51}-\rho_{ab}^{102}\right)\cdot ∆{t_{ab}}^{-1}+\left(\rho_{bc}^{51}-\rho_{bc}^{102}\right)\cdot ∆{t_{bc}}^{-1}+\left(\rho_{ac}^{51}-\rho_{ac}^{102}\right)\cdot ∆{t_{ac}}^{-1}\right]/3$$

(5) $$∆coh=\left[\left({cc}_{ab}^{51}-{cc}_{ab}^{102}\right)\cdot ∆{t_{ab}}^{-1}+\left({cc}_{bc}^{51}-{cc}_{bc}^{102}\right)\cdot ∆{t_{bc}}^{-1}+\left({cc}_{ac}^{51}-{cc}_{ac}^{102}\right)\cdot ∆{t_{ac}}^{-1}\right]/3$$

Following the extraction of ${∆NCP}_{w}$, ∆ins, and ∆coh, it is necessary to establish a quantitative relationship between these indicators and the fading bias itself. Since both intensity and coherence exhibit spatially and temporally varying sensitivities to fading bias, their relative contributions cannot be assumed to be constant across the scene or between different multilooking configurations. Therefore, instead of assigning empirical or fixed weights, we develop a data-driven weighting model that characterizes the joint influence of intensity and coherence variations on fading bias.

#### 2.2.2. Construction of a Data-Driven Pixel Weighting Function

To quantify the contribution of each pixel to the observed closure-phase variations, we model the empirical relationship between fading-bias-related phase deviations and two key indicators ∆ins and ∆coh using a multivariate linear formulation. This strategy allows the weighting process to adapt to spatially heterogeneous scattering environments and naturally incorporate the temporal characteristics of fading bias.

(1) Data normalization. Because the dynamic ranges of ${∆NCP}_{w}$, ∆ins, and ∆coh differ significantly, all variables are normalized to the interval [0, 1] using Min-Max scaling to ensure numerical stability and to avoid parameter bias.

(2) To capture the potentially coupled effects of ∆ins and ∆coh on fading bias-induced phase variations, we employ a multivariate linear regression model with an intercept and interaction term:

(6) $$∆{NCP}_{w}^{0}=a_{0}+a_{1}∆{ins}^{0}+a_{2}∆{coh}^{0}+a_{3}∆ins\cdot ∆coh$$

where $a_{1}$ and $a_{2}$ represent the primary contributions of amplitude and coherence variations; $a_{3}$ captures their interaction, reflecting the fact that fading bias is often jointly influenced by scattering strength and temporal decorrelation; $a_{0}$ is the intercept.

(3) Mapping regression output to a physically meaningful weight. Since the regression output is not restricted to [0, 1], its predicted value is transformed into a physically interpretable pixel weight using a sigmoid function:

(7) $$\omega =\frac{1}{1+exp({-∆NCPw}_{pred}^{0})}$$

This transformation ensures that the resulting pixel-wise weight satisfies 0<ω<1. ${NCPw}_{pred}^{0}$ represents the predicted normalized closure-phase deviation obtained from the multivariate linear regression model. It reflects the expected contribution of each pixel to the closure-phase noise given its intensity and coherence values, and serves as the input to the sigmoid function to generate the physically interpretable pixel weight ω. For each interferogram, the same normalization and regression model can be applied to obtain pixel-wise weights, which are then used in the weighted closure-phase SVD estimation.

#### 2.2.3. Bias Estimation Based on Weighted SVD

Given a dataset of N SAR images with spatial dimensions n×m, M interferograms are generated. From these, K triangle loops are formed, with each NCPw computed using Equation (1). Based on the learned pixel-wise weights, a diagonal weighting matrix C is constructed, and the corresponding weight matrix W is defined as:

(8) $$W=C^{T}C,\;\;\;\;\;\;C=W^{\frac{1}{2}}$$

A design matrix A is formulated based on triangle phase closure (TPC) relationships, and the unknown vector γ represents the fading bias for each interferogram at the pixel level. By incorporation the pixel-wise weights, the weighted design matrix and weighted observation vector are defined as:

(9) $$\tilde{A}=CA$$

(10) $$\tilde{b}\left(x,y\right)=C\varphi_{w}^{ncp}\left(x,y\right)$$

where $\varphi_{w}^{ncp}\left(x,y\right)$ denotes the NCPw observations at pixel location $\left(x,y\right)$.

The weighted design matrix is then decomposed via SVD:

(11) $$\tilde{A}=U\Sigma V^{T}$$

where U and V are orthogonal matrices, and Σ is a diagonal matrix containing the singular values of $\tilde{A}$.

The inversion is performed independently for each pixel $\left(x,y\right)$ using the Moore–Penrose pseudoinverse of $\tilde{A}$. The pseudoinverse $\Sigma^{+}$ id obtained by inverting the non-zero singular values in Σ. To improve numerical stability and suppress the influence of ill-conditioned components, singular values smaller than a predefined threshold are discarded during the inversion. The fading bias vector is then estimated as:

(12) $$\gamma \left(x,y\right)=\left(V\Sigma^{+}U^{T}\tilde{b}\left(x,y\right)\right)$$

The SVD-based inversion is performed independently for each pixel $\left(x,y\right)$. The resulting $\gamma \left(x,y\right)$ values are then assembled to form a two-dimensional fading bias field for each interferogram.

Finally, the estimated fading bias is removed from each interferogram to obtain the corrected interferogram stack:

(13) $$∆\phi_{i}^{c}=∆\phi_{i}-\gamma_{i}$$

where $∆\phi_{i}^{c}$ denotes the corrected phase of interferogram i.

### 2.3. Unwrapping Error Correction in Deformation Areas

After excluding low coherence points, rapid spatial variations caused by severe atmospheric delays and surface deformation are the primary factors leading to phase unwrapping errors [24,28]. NCPunw based correction methods require a sufficient number of correctly unwrapped interferograms as reliable references. For triangle loops without error compounding, single interferogram errors often associated with the longest temporal baselines allow the integer phase offset to be directly inferred from NCPunw. Correction proceeds by prioritizing such non-compounded loops, rapidly expanding the set of correctly unwrapped pixels and enabling subsequent correction of more complex cases. Moreover, these corrected interferograms provide reliable deformation-phase constraints, serving as a priori knowledge for precise correction in deforming regions. In this study, we integrate a sequential iterative strategy with deformation phase constraints to enable pixel-wise unwrapping error correction while addressing the impact of phase compounding. The detailed implementation is presented in the following sections.

#### 2.3.1. Initial High-Quality Phase Acquisition

The NCPunw of each triangle loop serves as the criterion for the selection of an initial high-quality phase set. A threshold λ of 1 rad is applied to the NCPunw values calculated from K triangle loops. The threshold was determined empirically based on statistical analysis of correctly unwrapped pixels. We found that, under normal phase gradients, the closure phase consistently remains below 1 rad, whereas pixels affected by unwrapping errors exhibit significantly larger deviations. This empirically derived threshold therefore provides a reliable criterion for separating valid closure phases from those contaminated by unwrapping errors. Pixels with NCPunw exceeding λ are masked to obtain the initial high-quality phase set, denoted as $\Psi_{optim}$. Although this initial set may include pixels affected by error compounding, the high quality phase set is iteratively refined, allowing such pixels to be progressively identified and corrected during subsequent iterations.

#### 2.3.2. Deformation Zone Localization and Reliable Deformation Information Extraction

Examination of pixels known to exhibit stable deformation behavior shows that their sign consistency defined as the proportion of interferograms sharing the same deformation sign typically exceeds 0.75, whereas pixels dominated by noise, atmospheric artifacts, or unwrapping uncertainties exhibit substantially lower consistency. Thus, κ=0.75 provides a robust and conservative criterion that effectively separates deformation dominated pixels from non-deformation pixels. This threshold ensures high reliability of the deformation mask while retaining sufficient spatial coverage for subsequent correction procedures.

(14) $$L=\frac{sum\left(sign\left(\Psi_{optim}^{i,j}\right)>\left(<\right)0\right)}{sum\left(\Psi_{optim}^{i,j}\right)}\;\;\;\;\;\;\;\left\{\begin{array}{c} L\geq 0.75,\left(i,j\right)\in defor\\ L<0.75,\left(i,j\right)\in non_{defor} \end{array}\right.$$

L denotes the final set of deformation pixels, and $\Psi_{optim}^{i,j}$ represents the initial high-quality phase list for a given pixel. Using the deformation area mask, the deformation phase series of each deformation pixel across all interferograms is extracted. Incorporating the temporal baselines ∆t, phase sets following the deformation trend ($\Psi_{defor}^{linear}$) are selected. $\Psi_{defor}^{linear}$ is the union of $\varphi_{defor(i,j)}^{linear}$. The mean of $\Psi_{defor}^{linear}$, calculated by Equation (15) as ${\bar{\Psi }}_{defor}^{linear}$, is used to replace the median value.

(15) $${\bar{\Psi }}_{defor}^{linear}=\frac{1}{n}\sum_{l=1}^{n}\varphi_{defor\left(i,j\right)}^{linear}$$

Using the median absolute deviation (MAD) method, the absolute deviation of each phase from ${\bar{\Psi }}_{defor}^{linear}$ calculated to obtain the set of absolute deviations $D_{i}$ (Equation (16)). Based on a threshold criterion (k= 3), outliers are identified and the phase values affected by compounding errors ($\varphi_{invalid}$) are further removed (Equation (17)). The resulting valid deformation phase set $\Psi_{defor}^{valid}$ is then used to reestimate integer phase ambiguities (Equation (18)).

(16) $$D_{i}=\left|\varphi_{i}-{\bar{\Psi }}_{defor}^{linear}\right|$$

(17) $$\varphi_{invalid}=\left\{\varphi_{i}|D_{i}>k\times MAD\right\}$$

(18) $$\Psi_{defor}^{valid}=\Psi_{defor}^{linear}-\Psi_{invalid}$$

#### 2.3.3. Re-Estimation of Integer Phase Ambiguities in Deformation Erroneous Zones

For NCPunw in triangle loops with unwrapping errors, two scenarios can be distinguished. In the first scenario, errors occur in only one interferogram, allowing direct correction using the corresponding triangle loop. In the second scenario, multiple interferograms contain simultaneous unwrapping errors, generating complex interactions that obscure true phase discontinuities. To address this, a sequential iterative correction strategy is applied. Closure triplets are ranked by coherence, inferred from temporal baselines and NCPunw values, and corrected in descending order. Each iteration updates the deformation estimates, gradually incorporating corrected pixels into the reliable phase set, which then contribute to subsequent corrections. For sufficiently redundant networks, this iterative procedure progressively converts compounded-error loops into effectively non-compounded cases, enabling robust unwrapping error correction in complex regions. The workflow comprises three main components:

(1) Calculate the sum of temporal baselines for each triangle loop, and use the closure phase residuals of triangle loop to derive the unwrapping nonzero closure phase set $\Psi^{NCPunw}$. These two parameters serve as sequential sorting references: $T^{tpc}$ as the primary (Equation (19)), $\Psi^{NCPunw}$ as the secondary (Equation (20)). The triangle loops are then sorted in ascending order to obtain a ranked sequence $sorted\left(\hat{\Psi }\right)$ (Equation (21)), which defines the correction order.

(19) $$T^{tpc}=\left\{\begin{array}{c} \left(t_{1,2}^{1}+t_{2,3}^{1}+t_{1,3}^{1}\right),\;\left(t_{2,3}^{2}+t_{3,4}^{2}+t_{2,4}^{2}\right),\;\cdots ,\;\\ \left(t_{a,b}^{k}+t_{b,c}^{k}+t_{a,c}^{k}\right) \end{array}\right\}$$

(20) $$\Psi^{NCPunw}=\left\{{TPC}_{NCP}^{1},\;{TPC}_{NCP}^{1},\;\cdots ,\;{TPC}_{NCP}^{k}\right\}$$

(21) $$S_{m}=\rho T_{m}^{tpc}+\Psi_{m}^{NCPunw}$$

where ρ is a weighting factor used to balance the relative contributions of the temporal baseline and the unwrapping-related closure phase residual. Both terms are normalized prior to combination to remove scale effects. Triangle loops are then sorted in ascending order of $S_{m}$. Loops with smaller scores are considered more reliable and are corrected first, whereas loops with larger scores, typically associated with longer temporal baselines and stronger unwrapping inconsistencies are deferred to later iterations. This ordering ensures that reliable deformation information is progressively accumulated and used to resolve more complex unwrapping error patterns.

(2) Identify a set of valid deformation phases with the same temporal baseline to estimate the integer phase ambiguity. If the interferogram to be corrected has a temporal span of $∆t_{A}$ and the erroneous phase is denoted as $\Psi_{defor}^{filtered}$, the valid deformation phases with the same temporal span, $\Psi_{defor}^{filtered}$, can be extracted from $\Psi_{defor}^{valid}$ (Equation (22)). The mean value $\varphi_{∆t_{A}}^{true}$ of $\Psi_{defor}^{filtered}$ (Equation (23)) is taken as the reference value for estimating the integer ambiguity k (Equation (24)). The resulting 2kπ is then applied to correct the errors in the original phase.

(22) $$\Psi_{defor}^{filtered}=\left\{\varphi_{i}\in \Psi_{defor}^{valid}|∆t_{\varphi_{i}}=∆t_{A}\right\}$$

(23) $$\varphi_{∆t_{A}}^{true}=\frac{1}{m}\sum_{i=i}^{m}\varphi_{defor\left(i\right)}^{filtered}$$

(24) $$k=round\left(\frac{\varphi_{∆t_{A}}^{true}-\varphi_{∆t_{A}}^{error}}{2\pi }\right)$$

(25) $$\varphi_{defor\left(i\right)}^{correct}=\varphi_{∆t_{A}}^{error}+2k\pi$$

(3) Recalculate the NPCunw of the triangle loops that include the corrected interferograms to assess the effectiveness of the correction. The successfully corrected pixels are continuously incorporated into the valid deformation phase set, enabling iterative refinement. This process is repeated until all abrupt phase errors are corrected, resulting in the final corrected interferogram set $\varphi_{defor\left(i\right)}^{correct}$ (Equation (25)).

**Figure 1:**
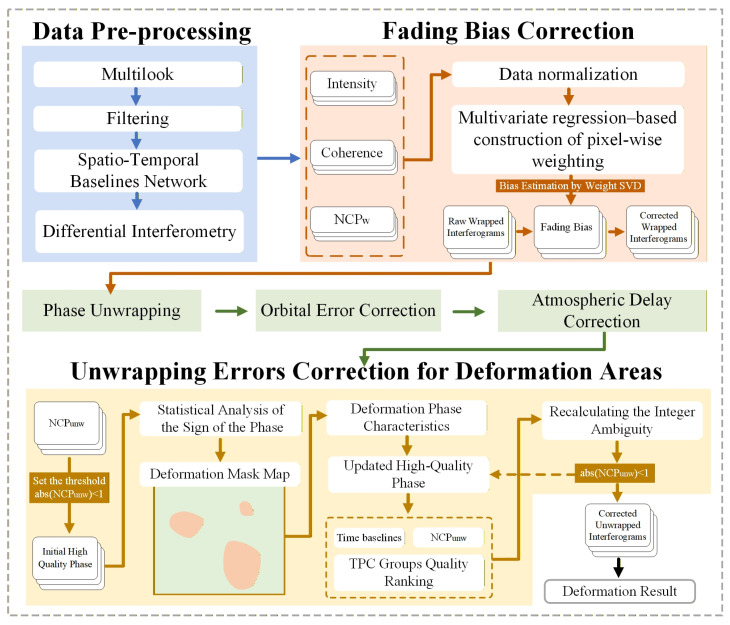
Processing workflow of the NCP-SBAS. node ${NCP}_{w}$ refers to the nonzero closure phase before unwrapping (fading bias); and node ${NCP}_{unw}$ indicates the nonzero closure phase after unwrapping (unwrapping errors).

The NCP-SBAS data processing workflow was established, as shown in Figure 1.

## 3. Study Area and Datasets

**Figure 2:**
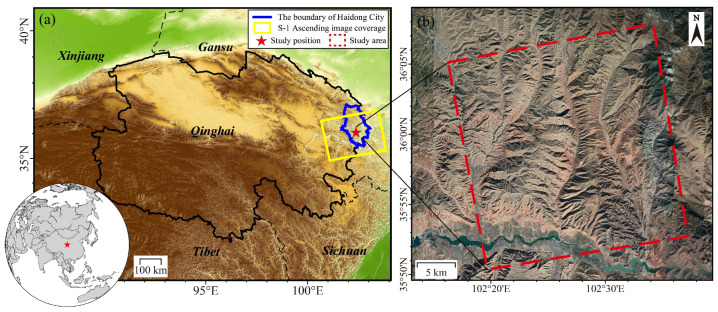
Location of the study area and data coverage. (**a**) The blue curve delineates the boundary of Haidong City; the yellow rectangle indicates the coverage of the Sentinel-1 data over the study area; the red pentagram marks the location of the study area; and (**b**) the red rectangle denotes the specific investigation area.

The study area is situated in the southern part of Hualong County, Qinghai Province, in northwestern China, within the transitional zone between the Qinghai–Tibet Plateau and the Loess Plateau (Figure 2). The region has an average elevation exceeding 2800 m and exhibits a typical plateau continental climate, with annual precipitation ranging from 300 to 500 mm, primarily occurring between June and September. The terrain is highly dissected, characterized by steep slopes, deeply incised valleys, and intense fluvial erosion. Quaternary loess deposits are present in portions of the area, which are prone to instability and exhibit high susceptibility to water-induced softening and collapse. In addition, extensive bedrock weathering has formed fractured zones. Due to these geomorphological and geological conditions, the region is highly susceptible to landslides, rendering it an ideal site for landslide monitoring and related geohazard studies. The fundamental data processing steps for the study area—including data preprocessing, multilook filtering, spatiotemporal network formation, differential interferometry, phase unwrapping, orbital error correction, atmospheric delay compensation, and SVD-based time-series inversion—were all performed using the GAMMA software (version 2.9).

**Figure 3:**
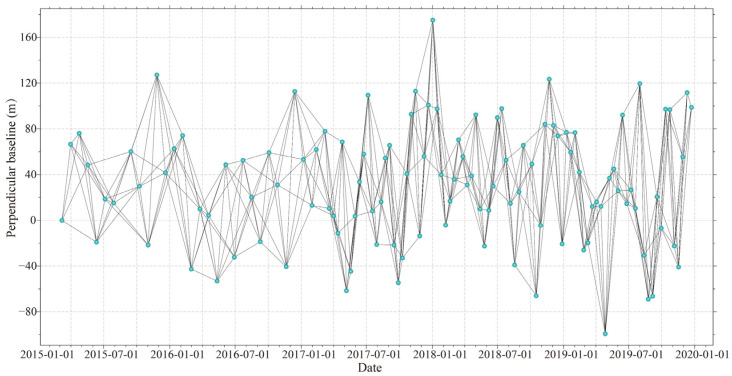
Spatial and temporal baselines of the selected Sentinel-1A pairs during the period from March 2015 to December 2019.

A total of 115 Sentinel-1 IW-mode single-look complex (SLC) images from 7 March 2015 to 23 December 2019 were acquired and organized into networks of consecutive four image sets (Figure 3), yielding 450 interferograms. A coherence threshold of 0.4 was applied, and phase unwrapping was conducted using the Minimum Cost Flow (MCF) algorithm. A multilook ratio of 10:2 was used for deformation inversion, while bias sensitivity estimation employed multilook ratios of 5:1 and 10:2. Goldstein filtering was applied for noise reduction. Monthly precipitation data for Haidong City were obtained from the National Tibetan Plateau Data Center (https://data.tpdc.ac.cn/, assessed on 15 July 2024).

## 4. Results and Analysis

### 4.1. Bias Sensitivity Analysis

**Figure 4:**
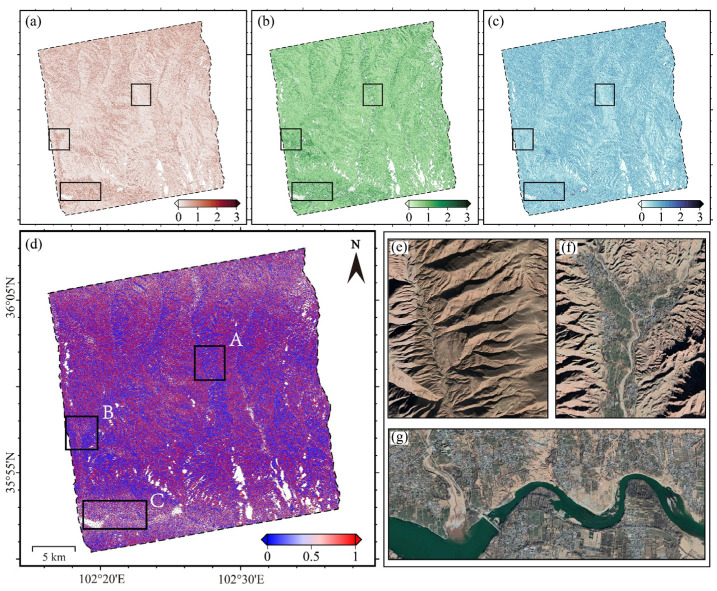
Bias Sensitivity Analysis. (**a**) coherence variations, ∆coh; (**b**) intensity variations, ∆ins; (**c**) NCPw differences, ∆NCPw; (**d**) Bias sensitivity map (0–1 scale), where red (approaching to 1) indicate intensity-dominated bias, and blue (approaching to 0) reflects coherence-dominated bias; (**e**–**g**) Optical images corresponding to the marked regions in (**d**) representing bare rock (A), cultivated land (B), and human activity (C), respectively.

Using three wrapped interferograms (denoted as 20150307_20150331, 20150331_20150424, and 20150307_20150424) as an illustrative example, we analyze the relative contributions of amplitude and coherence variations to the three-interferogram NCPw at the pixel level. A pixel-wise sensitivity index S is defined as:

(26) $$S=\frac{a_{1}\cdot ∆{ins}^{0}}{a_{1}\cdot ∆{ins}^{0}+a_{2}\cdot ∆{coh}^{0}}$$

where $a_{1}$ and $a_{2}$ are the regression coefficients corresponding to Δins and Δcoh, respectively. By construction, S→0 indicates that the closure phase is predominantly influenced by coherence variations, while S→1 indicates that amplitude variations are the dominant factor. Finally, the bias sensitivity map was computed using Equation (26), as illustrated in Figure 4d. According to Figure 4, ${∆NCP}_{w}$ (Figure 4a) and ∆cho (Figure 4c) exhibit a generally positive correlation at the regional scale. Larger ∆cho values are typically associated with more pronounced variations in ${∆NCP}_{w}$. Since ∆coh is negatively correlated with interferometric coherence, fading bias tends to increase as coherence decreases. This finding is consistent with the results reported by [30]. Distinct sensitivity differences among various surface features can be observed in Figure 4d, allowing for clear delineation of land-cover boundaries.

Three representative regions were selected for further analysis, as indicated by the black solid boxes (A, B and C) in Figure 4d. Region A corresponds to a largely unvegetated mountainside. Despite noticeable amplitude variations, it exhibits relatively low fading bias and minor changes in coherence. Appearing in blue in Figure 4d, this region is minimally affected by fading bias, indicative of high pixel quality and a strong correlation between bias and coherence. Region B is an agricultural area, where the spatial distributions of fading bias and amplitude variation are closely aligned, as clearly seen in Figure 4a,b. Coherence changes are moderate. The region appears primarily in red in Figure 4d, suggesting a strong dependence of fading bias on amplitude variation. Region C is located near a watershed with intensive human activity. It shows large amplitude fluctuations and significant coherence degradation, along with the highest bias levels. This highly unstable region appears light purple in Figure 4d, near the median sensitivity level, indicating that fading bias here is influenced by both amplitude variation and coherence.

### 4.2. InSAR Deformation Results and Analysis

**Figure 5:**
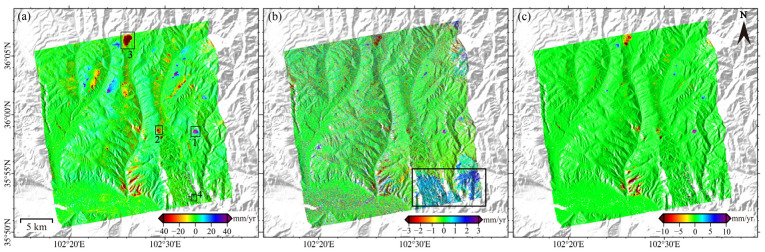
Deformation results derived from SBAS and NCP-SBAS. (**a**) Average deformation rate after NCP correction; (**b**) Error caused by fading bias. The black rectangular box delineates the area with pronounced local fading bias; (**c**) Error caused by unwrapping errors. The numbered rectangular areas will be analyzed in detail in the following sections.

We obtained the mean deformation rates after (Figure 5a) NCP correction. In addition, we quantified the errors arising from fading bias (Figure 5b) and from unwrapping errors (Figure 5c). The combined effect of these error components equals the total correction impact.

**Figure 6:**
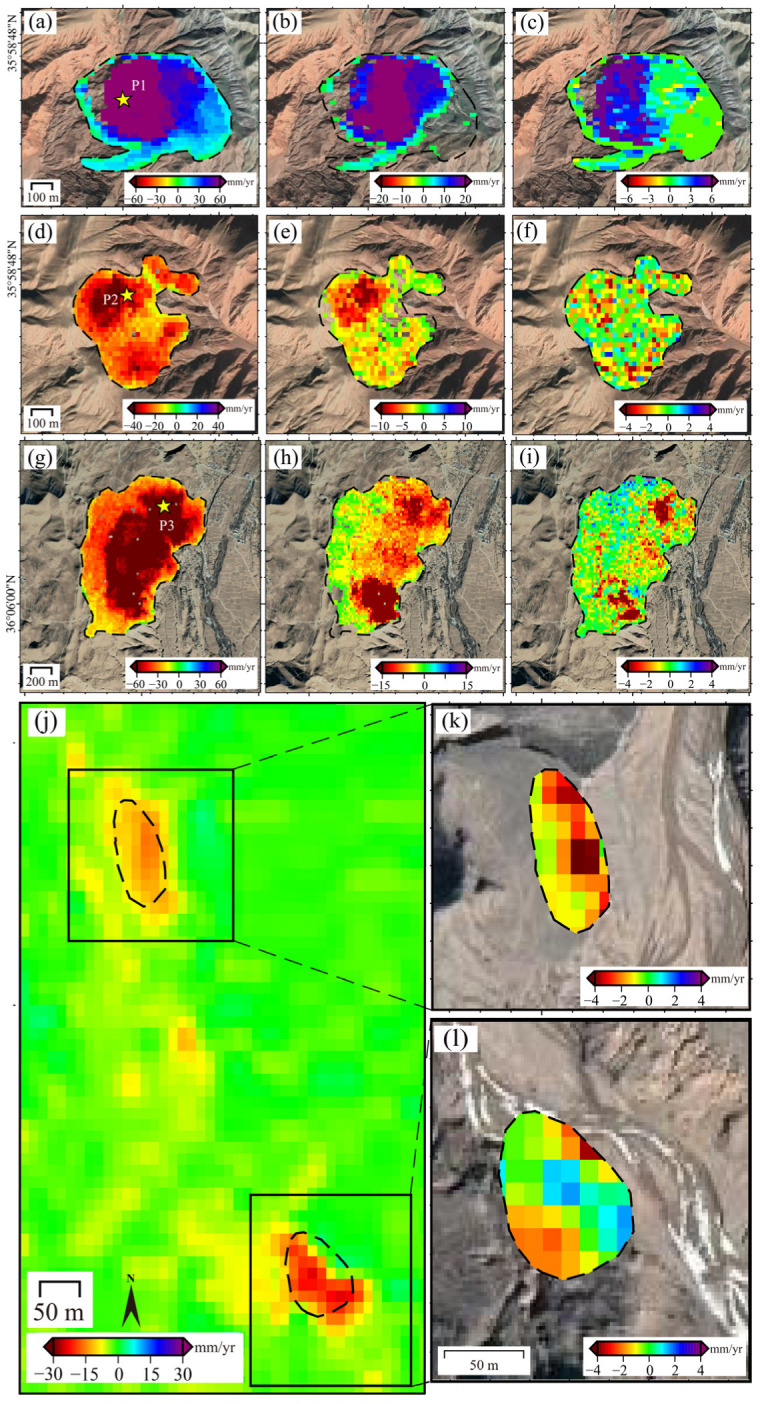
Deformation of the four areas in Figure 5 and their fading biases and unwrapping errors. Panels (**a**–**c**) correspond to area 1, (**d**–**f**) correspond to area 2; and (**j**–**l**) to Area 4, with (**j**) representing landslides in area 4, and (**g**–**i**) providing detailed views of these landslides.

The spatial distribution of deformation signals remains largely consistent before and after correction. However, significant deformation zones demonstrate systematic underestimation in uncorrected results. Figure 5c further reveals that unwrapping errors typically lead to underestimated displacement rates. The maximum difference before and after correcting unwrapping errors reaches up to 8.5 cm/yr, with an average difference of 34 mm/yr. As shown in Figure 6b, the fading bias exhibits significant spatial variability. The maximum difference before and after bias correction reaches 7.12 mm/yr, with an average difference of 1.02 mm/yr.

Two main regions are notably affected by fading bias: the area within the solid rectangular box in Figure 6b, and the deformation zone. The area in the box shows pronounced systematic bias, manifesting as deformation signal overestimation. This results from steep mountainous terrain and complex scattering mechanisms that amplify spatiotemporal backscattering inconsistencies. In the deformation zone, fading biases are concentrated in regions with large deformation magnitudes and those along deformation boundaries. The bias magnitude is proportional to the deformation level. Four representative landslide areas, labeled 1, 2, 3 and 4 were selected for detailed analysis (Figure 5a). For each area, we extracted the average deformation velocity along with the corresponding corrections for fading bias and unwrapping errors.

As no unwrapping errors were detected in Area 4, its unwrapping errors are not shown in Figure 6. Both fading bias and unwrapping errors are predominantly distributed in regions with larger deformation magnitudes (Figure 6a–j). Additionally, fading bias are often concentrated along the deformation zone edges. For areas with minor overall bias trends (outside the solid-line rectangle), the bias inside deformation zones aligned with the displacement direction, resulting in fading bias underestimation. As shown in Figure 6k, in regions with significant overall bias trends (outlined by the solid-line rectangle in Figure 5b), the bias magnitude correlates with deformation magnitude. In one deformation zone, large-deformation regions exhibit biases align with the deformation direction, whereas small-deformation regions have biases aligned with the overall bias trend.

**Figure 7:**
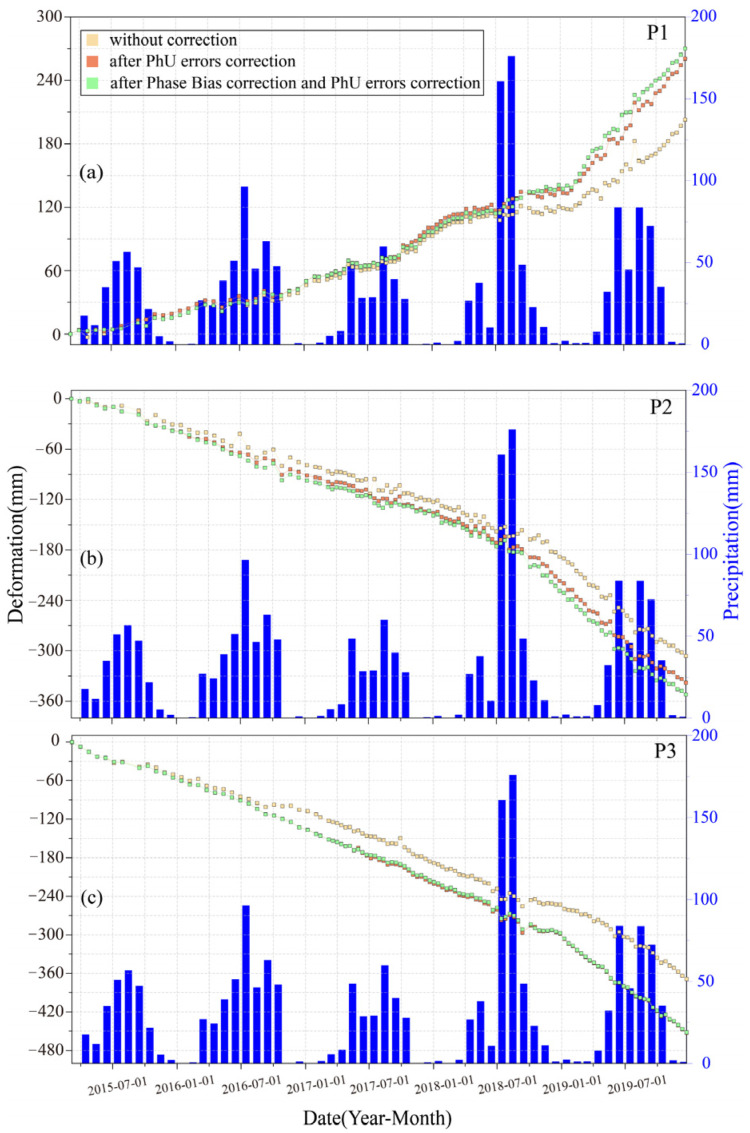
Deformation time series overlaid with monthly precipitation. (**a**) Point 1; (**b**) Point 2; and (**c**) Point 3 (locations shown in Figure 6).

Based on the cumulative displacement statistics within the deformation zones, the average difference in cumulative displacement is 5.1 cm, with a maximum difference reaching 15 cm. To further investigate the time series, we selected three representative ponit1, point2 and point3 in Figure 6a,d,g. For each point, we analyzed three displacement time series: (1) uncorrected raw data, (2) unwrapping error corrected only, and (3) fully corrected (both unwrapping errors and fading bias). As shown in Figure 7, unwrapping errors result in deformation underestimation. The influence of fading bias varies across regions. At point3 (negligible fading bias), the corrected and uncorrected time series exhibit minimal fluctuations, and the final cumulative displacements are nearly identical. This indicates that unwrapping errors are the primary error source at this point. Both point1 and point2 are affected by fading bias, with before and after cumulative displacement differences of 14 mm and 8 mm, respectively. After correction, the fading bias leads to underestimation. In addition, we obtained the monthly average precipitation data for the study area in March 2015 from to December 2019. A sharp increase in rainfall occurred in July and August 2018, which triggered accelerated deformation rates (Figure 7). Notably, both unwrapping errors and fading biases became more pronounced following the rainfall event.

## 5. Discussion

### 5.1. In Effectiveness of Sequential Iteration for Unwrapping Errors Correction in Error Compounding Regions

**Figure 8:**
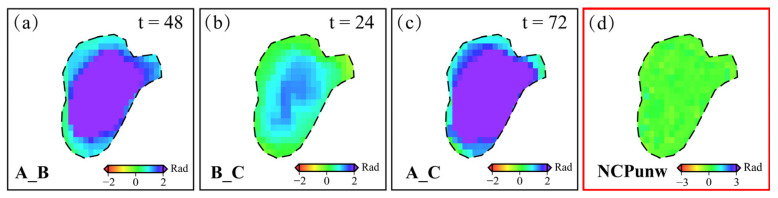
Schematic illustration of a triangle loop exhibiting consistent deformation and phase consistency.

When correct, unwrapping errors in deformation zones, ${NCP}_{unw}$ serve two key functions: identifying areas of discontinuity prior to correction, and validating the correction effectiveness. As shown in Figure 8, in regions with either corrected or inherently absent unwrapping errors, the three interferograms forming a triangle loop maintain consistent deformation, resulting ${NCP}_{unw}$ approaching zero.

In triangle loops affected by phase unwrapping errors, longer temporal baselines are generally associated with lower coherence and larger deformation magnitudes. In a triangle loop with relatively short average temporal baselines, unwrapping errors are most likely to occur in the interferogram with the longest baseline [29]. However, longer temporal baselines and reduced coherence lead to increasingly complex unwrapping errors patterns. When two of the three images contain same-sign errors, unwrapping error compounding may occur, whereby the phase errors cancel out during the triangle loop computation. In such cases, the ${NCP}_{unw}$ fails to capture the actual unwrapping error, leading to incorrect localization of phase errors and significantly compromising the correction accuracy. Therefore, sequential and iterative correction strategies are essential for effective unwrapping errors mitigation. To investigate the necessity of sequential iteration for correcting unwrapping discontinuities in deformation zones, we analyzed a representative area using two triangle loops: ① 20161120_20170107, 20170107_20170131, and 20161120_20170131; and ② 20161120_20170131, 20170131_20170212, and 20161120_20170212. For clarity, letters are used to denote interferogram IDs. Group ① includes AB, BC, and AC; Group ② includes AC, CD, and AD.

**Figure 9:**
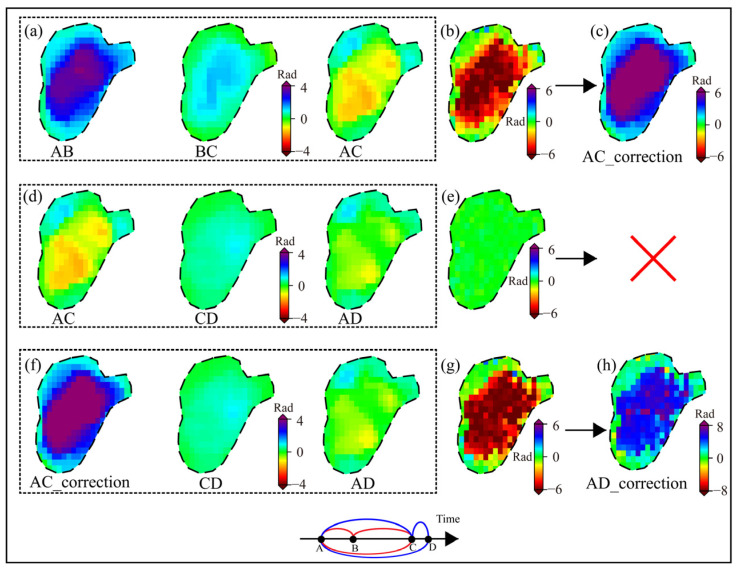
Sequential correction of unwrapping errors. (**a**) Triangle loop without compounding and (**b**) its ${NCP}_{unw\_a}$; (**d**) Triangle loop affected by compounding before correction of interferogram AC and (**e**) its ${NCP}_{unw\_b\;raw}$; (**f**) Triangle loop after AC correction and its (**g**) ${NCP}_{unw\_b\;corr}$. (**c**,**h**) show the corrected unwrapping errors for interferograms AC and AD, respectively. Circular markers indicate unwrapping errors (red: present, green: absent).

We extracted unwrapped interferograms from Groups ① and ②, with orbital and atmospheric errors corrected. Figure 9a show group ① and Figure 9d show group ②. In Group ①, no compounding occurs. The phase discontinuity appears in AC, which has the longest temporal baseline. The computed ${NCP}_{unw\_a}$ (Figure 9b) enables accurate localization of the error region. In contrast, Group ② exhibits aliasing, as both AC and AD experience unwrapping error with the same sign. These errors cancel out during ${NCP}_{unw\_b\;raw}$ calculation, as shown in Figure 9e, preventing effective identification of the unwrapping errors. Specifically, the phase unwrapping errors in AC can be corrected using the method described in Section 2.3, yielding a corrected interferogram AC_corr (Figure 9c). This corrected interferogram is then included in the high-quality dataset and contributes to the correction of Group ②, as illustrated in Figure 9f. In this case, compounding is eliminated, and the ${NCP}_{unw\_b\;corr}$ result (Figure 9g) successfully identifies the unwrapping error region in AD. This iterative correction continues in a similar manner, effectively mitigating the impact of compounding on unwrapping error correction. For the landslide in Area 3 of Figure 6a, the deformation zone was treated as an integrated unit for correction. Both non-sequential and sequential iterative strategies were applied. Among the 450 interferograms, 181 phase discontinuities were detected. The non-sequential correction strategy addressed only 88 errors, while the sequential iterative strategy successfully corrected 157.

### 5.2. Comparison with Other Fading Bias Correction Method and Validation of Effectiveness

To further validate the effectiveness of the proposed method, we compared the fading bias correction performance of wTPC with the TVm (Time Variant model) [22]. The TVm adopts a 72 day temporal window for bias estimation and correction. Under error-free conditions, deformation time series derived from the same set of SAR images, regardless of different spatiotemporal baseline networks, should be consistent. Therefore, any discrepancy in the results obtained from different baseline configurations can serve as an indicator of correction performance.

**Figure 10:**
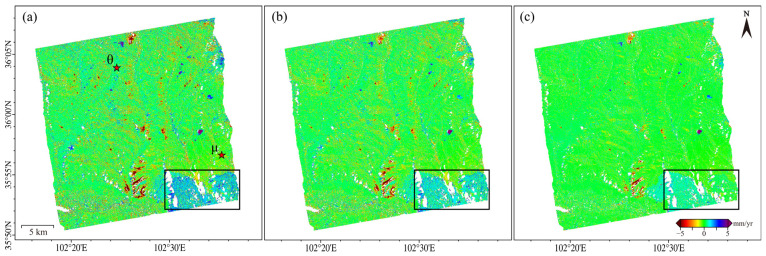
Deformation differences across baseline combinations: (**a**) uncorrected results; (**b**) TVm-corrected results; (**c**) wTPC-corrected results. The pentagram markers in each subplot denote the points (θ and μ) selected for subsequent time-series analysis. The black rectangular box delineates the area with pronounced local fading bias.

In this study, we conducted deformation time series analysis using two baseline configurations: the full baseline set, constructed with connections to the subsequent four images, and a long baseline subset, obtained by removing interferometric pairs with temporal baselines shorter than 36 days from the full set. Differences in the estimated average deformation rates between the two configurations are shown in Figure 10. To isolate the specific contribution of fading bias correction, this comparative analysis intentionally excluded phase unwrapping error correction. Overall, the mean deformation rate differences are 1.09 mm/yr, 0.91 mm/yr, and 0.62 mm/yr, respectively, demonstrating the superior performance of the wTPC approach.

**Figure 11:**
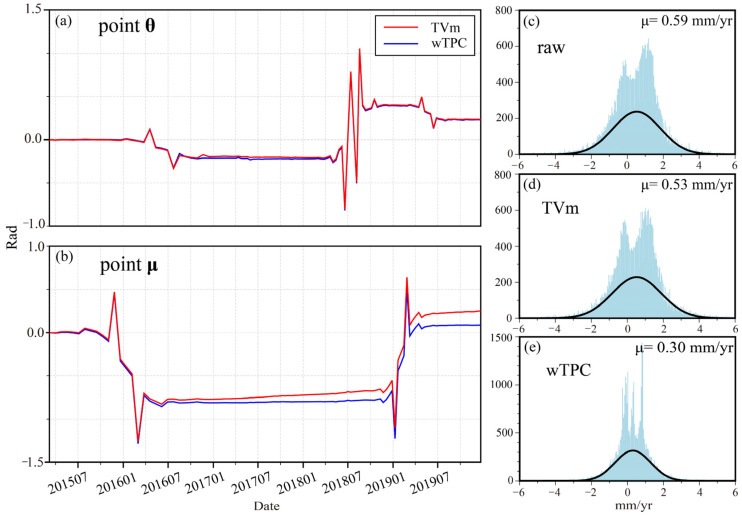
Time series of fading biases at points θ (**a**) and μ (**b**) after correction using TVm and wTPC, along with histograms of deformation rate differences within the rectangular region in Figure 10. (**c**) uncorrected results; (**d**) TVm-corrected results; (**e**) wTPC-corrected results.

To evaluate temporal consistency, we selected two representative points (θ and μ) where both wTPC and TVm methods demonstrated effective correction. Figure 11a,b show the fading bias time series, derived by differencing with the original deformation data. While both methods exhibit strong temporal agreement, the TVm method shows reduced effectiveness in active deformation zones, and areas with pronounced local bias trends. To further assess the correction performance in such areas, we conducted a statistical analysis on the region outlined by the black rectangle in Figure 10, where significant bias trends were observed. The histograms of deformation rate differences for the raw, TVm-corrected, and wTPC-corrected results are shown in Figure 11c, Figure 11d, and Figure 11e, respectively. The corresponding mean differences are 0.59 mm/yr, 0.51 mm/yr, and 0.30 mm/yr, confirmed that wTPC achieves the best correction performance. This improvement is likely due to the underlying mechanisms of the two methods. The TVm model estimates systematic bias primarily based on temporal baselines, with limited consideration of spatial characteristics. In contrast, wTPC incorporates both the spatial sensitivity of fading bias and its temporal fading behavior, enabling a more comprehensive spatiotemporal correction.

**Figure 12:**
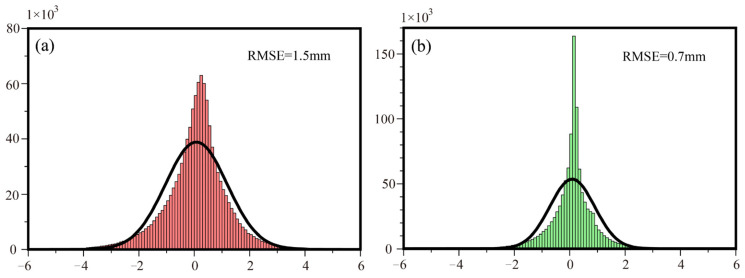
Validation Histograms. (**a**) Statistical histogram of the non-deforming area before correction (depicted in red); (**b**) Statistical histogram of the non-deforming area after correction (depicted in green).

In addition, the effectiveness of the proposed method is further validated and evaluated by comparing deformation estimates obtained from different baseline combinations. A deformation mask threshold is determined based on the standard deviation of the deformation results, and deformation areas are subsequently masked out using this threshold. The effectiveness of the correction is then assessed by analyzing the statistical characteristics of the non-deforming areas. Specifically, the statistical analyses of the non-deformation regions in Figure 10a,c are presented, with the corresponding histogram distributions and RMSE values shown in Figure 12. The results indicate that the RMSE of the deformation estimates obtained using the proposed method is 0.7 mm, compared to 1.5 mm for the original deformation results, corresponding to an overall accuracy improvement of approximately 53%.

### 5.3. Impacts of Fading Bias on Landslide Detection

Fading bias introduces systematic errors with pronounced spatial heterogeneity, which may lead to misestimation of the spatial distribution and magnitude of landslide deformation. For slowly moving or low-magnitude landslides, the deformation signal may be obscured by the bias. Therefore, this section conducts landslide identification within the study area and assesses the impacts of fading bias on detection. Landslide identification was conducted through visual interpretation of the deformation fields before and after ${NCP}_{w}$ correction, assisted by Google Earth.

**Figure 13:**
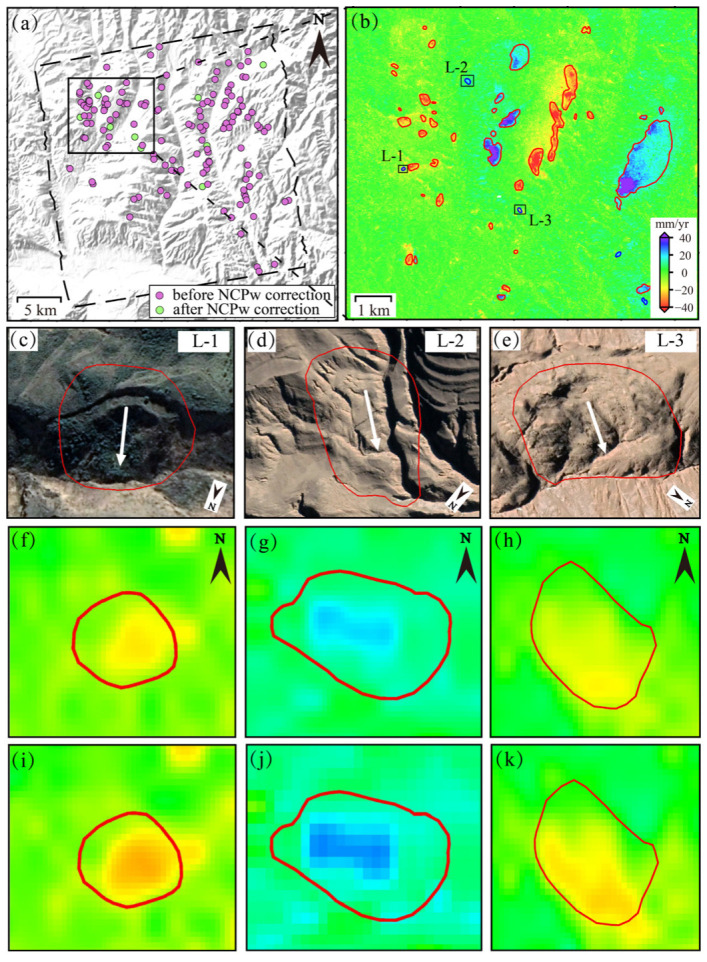
Impact of fading bias on landslide detection. (**a**) detection differences before (purple) and after (green) bias correction; (**b**) Zoomed deformation map of the black boxed area in (**a**) (red: consistently detected; blue: newly identified landslides); (**c**–**k**) correspond to the corrected and uncorrected deformation fields and optical images of the newly identified landslides L-1, L-2 and L-3 within the boxed area in (**b**). (Dashed box: study area. Red circle: landslide boundary derived from deformation results. White arrow: sliding direction.)

A total of 108 landslides were detected before correction, primarily distributed along mountain ridges and riverbanks. After correction, 118 landslides were identified, including 10 newly recognized landslides (Figure 13a). These newly detected sites exhibit relatively small deformation extent and magnitude, with an average area of 13,775 m^2^ and a mean deformation rate of 1.61 mm/yr. To illustrate the improvement in landslide detection, a representative area outlined by the rectangle in Figure 13a is selected for detailed analysis (Figure 13b). Within this area, five new landslides were detected. Among them, three landslides (labeled L-1, L-2 and L-3 in Figure 13b) were selected for further analysis. The deformation fields before and after correction, along with corresponding optical imagery, are shown in Figure 13c–k, respectively. All the three landslides exhibit clear linear cracks along the rear or lateral margins of the hillslopes within the delineated boundaries in the optical images, consistent with typical visual features of landslides. Before fading bias correction, the deformation magnitudes are often comparable to the background noise level. These scattered and spatially limited signals are difficult to confidently identified as landslides. After bias correction, deformation signals exhibit significantly enhanced spatial coherence and recognizability.

## 6. Conclusions

This study proposes an improved SBAS-InSAR processing method that accounts for NCP. Correction strategies for fading biases and unwrapping errors in deformation areas are introduced to enhance phase consistency throughout the inversion process. This approach enables retrieve more accurate deformation fields and allows quantitatively assess the effects of fading biases on landslide detection and analysis. The key contributions of this work are as follows: (1) A weighted SVD-based method is proposed for fading bias correction. The approach first constructs a pixel-wise weighting model using amplitude and coherence information. Finally, ${NCP}_{w}$ is calculated based on triangle loops to achieve bias correction considering both spatial and temporal variability. (2) An iterative optimization based algorithm is developed to correct unwrapping errors in deformation areas. The method first identifies potential unwrapping errors and selects an initial set of high-quality interferograms by ${NCP}_{unw}$, on which basis deformation zones are accurately localized. Leveraging reliable deformation signals and a sequential iterative strategy, a pixel-wise correction model is established to address unwrapping errors, including cases affected by compounding. After error correction, the number of identified landslides increased from 108 to 118, corresponding to an improvement of approximately 9.3% in landslide detection capability. Compared with the uncorrected result and TVm, the proposed method reduces the mean difference from 0.59 mm/yr and 0.51 mm/yr to 0.30 mm/yr, corresponding to error reductions of approximately 49.2% and 41.2%, respectively. In terms of the differences in deformation results under different baseline combinations, the proposed method improves the accuracy by 53%. One limitation of this study lies in the ${NCP}_{w}$ estimation model, which does not yet fully account for seasonal variations, potentially introducing uncertainties in long-term monitoring. Moreover, the correction of unwrapping errors in deformation areas relies on the temporal consistency of deformation trends. When the deformation exhibits alternating uplift and subsidence, a more comprehensive deformation model is required to support accurate correction.

## Data Availability

Deformation data associated with this article can be obtained from http://insarclub.com.

## References

[1] Bekaert D.P.S., Handwerger A.L., Agram P., Kirschbaum D.B. (2020). InSAR-Based Detection Method for Mapping and Monitoring Slow-Moving Landslides in Remote Regions with Steep and Mountainous Terrain: An Application to Nepal. Remote Sens. Environ..

[2] Chaussard E., Wdowinski S., Cabral-Cano E., Amelung F. (2014). Land Subsidence in Central Mexico Detected by ALOS InSAR Time-Series. Remote Sens. Environ..

[3] Yan Y., Doin M.-P., Lopez-Quiroz P., Tupin F., Fruneau B., Pinel V., Trouve E. (2012). Mexico City Subsidence Measured by InSAR Time Series: Joint Analysis Using PS and SBAS Approaches. IEEE J. Sel. Top. Appl. Earth Obs. Remote Sens..

[4] Roy P., Martha T.R., Khanna K., Jain N., Kumar K.V. (2022). Time and Path Prediction of Landslides Using InSAR and Flow Model. Remote Sens. Environ..

[5] Yang Z., Li Z., Zhu J., Wang Y., Wu L. (2020). Use of SAR/InSAR in Mining Deformation Monitoring, Parameter Inversion, and Forward Predictions: A Review. IEEE Geosci. Remote Sens. Mag..

[6] Ferretti A., Prati C., Rocca F. (2001). Permanent Scatterers in SAR Interferometry. IEEE Trans. Geosci. Remote Sens..

[7] Hooper A. (2008). A Multi-temporal InSAR Method Incorporating Both Persistent Scatterer and Small Baseline Approaches. Geophys. Res. Lett..

[8] Lanari R., Mora O., Manunta M., Mallorquí J.J., Berardino P., Sansosti E. (2004). A Small-Baseline Approach for Investigating Deformations on Full-Resolution Differential SAR Interferograms. IEEE Trans. Geosci. Remote Sens..

[9] Ferretti A., Prati C., Rocca F. (2000). Nonlinear Subsidence Rate Estimation Using Permanent Scatterers in Differential SAR Interferometry. IEEE Trans. Geosci. Remote Sens..

[10] Ferretti A., Fumagalli A., Novali F., Prati C., Rocca F., Rucci A. (2011). A New Algorithm for Processing Interferometric Data-Stacks: SqueeSAR. IEEE Trans. Geosci. Remote Sens..

[11] Chen Y., Tong Y., Tan K. (2020). Coal Mining Deformation Monitoring Using SBAS-InSAR and Offset Tracking: A Case Study of Yu County, China. IEEE J. Sel. Top. Appl. Earth Obs. Remote Sens..

[12] Li S., Xu W., Li Z. (2022). Review of the SBAS InSAR Time-Series Algorithms, Applications, and Challenges. Geod. Geodyn..

[13] Yao J., Yao X., Liu X. (2022). Landslide Detection and Mapping Based on SBAS-InSAR and PS-InSAR: A Case Study in Gongjue County, Tibet, China. Remote Sens..

[14] Dong J., Niu R., Li B., Xu H., Wang S. (2023). Potential Landslides Identification Based on Temporal and Spatial Filtering of SBAS-InSAR Results. Geomat. Nat. Hazards Risk.

[15] Jennison R.C. (1958). A Phase Sensitive Interferometer Technique for the Measurement of the Fourier Transforms of Spatial Brightness Distributions of Small Angular Extent. Mon. Not. R. Astron. Soc..

[16] De Zan F., Zonno M., Lopez-Dekker P. (2015). Phase Inconsistencies and Multiple Scattering in SAR Interferometry. IEEE Trans. Geosci. Remote Sens..

[17] Gu X., Li Y., Zuo X., Bu J., Yang F., Yang X., Li Y., Zhang J., Huang C., Shi C. (2024). Image Compression–Based DS-InSAR Method for Landslide Identification and Monitoring of Alpine Canyon Region: A Case Study of Ahai Reservoir Area in Jinsha River Basin. Landslides.

[18] Michaelides R.J., Zebker H.A., Zheng Y. (2019). An Algorithm for Estimating and Correcting Decorrelation Phase from InSAR Data Using Closure Phase Triplets. IEEE Trans. Geosci. Remote Sens..

[19] Maghsoudi Y., Hooper A.J., Wright T.J., Lazecky M., Ansari H. (2022). Characterizing and Correcting Phase Biases in Short-Term, Multilooked Interferograms. Remote Sens. Environ..

[20] Ma Z., Wang N., Yang Y., Aoki Y., Wei S. (2025). Unwrapping Error and Fading Signal Correction on Multi-Looked InSAR Data. ISPRS J. Photogramm. Remote Sens..

[21] Zheng Y., Fattahi H., Agram P., Simons M., Rosen P. (2022). On Closure Phase and Systematic Bias in Multilooked SAR Interferometry. IEEE Trans. Geosci. Remote Sens..

[22] Falabella F., Pepe A. (2022). On the Phase Nonclosure of Multilook SAR Interferogram Triplets. IEEE Trans. Geosci. Remote Sens..

[23] Sica F., Calvanese F., Scarpa G., Rizzoli P. (2022). A CNN-Based Coherence-Driven Approach for InSAR Phase Unwrapping. IEEE Geosci. Remote Sens. Lett..

[24] Biggs J., Wright T., Lu Z., Parsons B. (2007). Multi-Interferogram Method for Measuring Interseismic Deformation: Denali Fault, Alaska. Geophys. J. Int..

[25] Wu S., Zhang B., Ding X., Shahzad N., Zhang L., Lu Z. (2022). A Hybrid Method for MT-InSAR Phase Unwrapping for Deformation Monitoring in Urban Areas. Int. J. Appl. Earth Obs. Geoinf..

[26] Yang X., Wang H., Pagli C., Ng A.H.-M., He Q. (2024). Closure-Based Correction of InSAR Phase Unwrapping Errors by Integrating Block-Wise Tikhonov Regularization and Flux Analysis. IEEE Trans. Geosci. Remote Sens..

[27] Yunjun Z., Fattahi H., Amelung F. (2019). Small Baseline InSAR Time Series Analysis: Unwrapping Error Correction and Noise Reduction. Comput. Geosci..

[28] Benoit A., Pinel-Puysségur B., Jolivet R., Lasserre C. (2020). CorPhU: An Algorithm Based on Phase Closure for the Correction of Unwrapping Errors in SAR Interferometry. Geophys. J. Int..

[29] Liu Y., Wu H., Zhang Y., Lu Z., Kang Y., Wei J. (2025). 3D Automatic Detection and Correction for Phase Unwrapping Errors in Time Series SAR Interferometry. ISPRS J. Photogramm. Remote Sens..

[30] Zwieback S., Liu X., Antonova S., Heim B., Bartsch A., Boike J., Hajnsek I. (2016). A Statistical Test of Phase Closure to Detect Influences on DInSAR Deformation Estimates Besides Displacements and Decorrelation Noise: Two Case Studies in High-Latitude Regions. IEEE Trans. Geosci. Remote Sens..

[31] De Zan F., Gomba G. (2018). Vegetation and Soil Moisture Inversion from SAR Closure Phases: First Experiments and Results. Remote Sens. Environ..

